# Risk Factors Analysis and Prediction Model Establishment of Intestinal Metaplasia or Dysplasia in Patients With Chronic Atrophic Gastritis: A Multi-Center Retrospective Study

**DOI:** 10.3389/fmed.2022.912331

**Published:** 2022-05-18

**Authors:** Bei Pei, Ziang Wen, Qi Yang, Jieyu Wang, Qinglin Cao, Longfei Dai, Xuejun Li

**Affiliations:** ^1^The Graduated School, Anhui University of Traditional Chinese Medicine, Hefei, China; ^2^The Graduated School, Anhui Medical University, Hefei, China; ^3^Department of Gastroenterology, The Second Affiliated Hospital of Anhui University of Traditional Chinese Medicine, Hefei, China; ^4^Department of Gastrointestinal Surgery, The First Affiliated Hospital of Anhui Medical University, Hefei, China

**Keywords:** chronic atrophic gastritis, intestinal metaplasia, risk factors, prediction model, dysplasia

## Abstract

**Objective:**

To investigate the risk factors and construct a prediction model of chronic atrophic gastritis (CAG) patients with intestinal metaplasia or dysplasia.

**Method:**

The clinical data of 450 patients with CAG who were diagnosed and treated in the Department of Gastroenterology of the Second Affiliated Hospital of Anhui University of Traditional Chinese Medicine from June 2016 to February 2022 were collected. Single and multiple factors logistic regression analysis were used to explore the risk factors of intestinal metaplasia or dysplasia in patients of training cohort. Then, we constructed a model to predict the onset of intestinal metaplasia or dysplasia based on the data of training cohort, following which we tested the model in an external validation cohort of 193 patients from a local university teaching hospital. The ROC curve, calibration curve, and decision curve analysis were used to evaluate the accuracy of the prediction model.

**Result:**

*Helicobacter pylori* (*H. pylori*, HP) infection, pepsinogen I, gastrin-17, and the number of lesions were found to be independent rick factors of the model. The liner prediction model showed excellent predictive value in both training cohort and validation cohort.

**Conclusion:**

HP infection, pepsinogen I, gastrin-17, and the number of lesions are independent risk factors for intestinal metaplasia or dysplasia in patients with CAG. The prediction model constructed based on these factors has a high accuracy and excellent calibration, which can provide a great basis for condition assessment and individualized treatment of the patients.

## Introduction

Chronic atrophic gastritis (CAG) is a common gastric disease characterized by a decreased number of gastric mucosa and mucosal atrophy, with or without intestinal metaplasia or dysplasia. The patients often have some non-specific symptoms, such as fullness and pain in the upper abdomen, and loss of appetite and belching (1). CAG is a precancerous state of gastric cancer, the pathogenesis of which is not yet clear. However, the theory of a multi-stage, multi-step phase suggested by Correa is widely accepted now (2). The gastric mucosa of patients with CAG is constantly damaged by a chronic inflammatory, which leads to the occurrence of intestinal metaplasia or dysplasia and consequently to the development of gastric cancer. CAG is associated with intestinal gastric cancer, with patients having an annual chance of developing gastric cancer of about 0.1%, and the presence of precancerous lesions increases the risk of gastric cancer (3). Therefore, CAG is a key point in the prevention of gastric cancer, and early diagnosis and timely treatment of the disease during this period can reduce the incidence of gastric cancer. In this study, we retrospectively analyzed the clinical information and examination data of patients with CAG treated at our hospital to investigate the factors influencing the development of intestinal metaplasia or dysplasia in patients with CAG, and a predictive model was also constructed based on the findings. This study may provide a basis for future research on the prevention, diagnosis, and treatment of the disease and its complications.

## Materials and Methods

### Selection of Research Participants

Data of 485 patients who were diagnosed with CAG at the Second Affiliated Hospital of Anhui University of Traditional Chinese Medicine from June 2016 to February 2022 were collected. The patients who did not fulfill the inclusion criteria were excluded. Finally, 450 patients with CAG were identified as training cohort. According to the same criteria, 193 patients from the First Affiliated Hospital of Anhui Medical University were identified as validation cohort. The diagnosis of CAG was confirmed by gastroscopy and pathology. Based on the results, all patients in training and validation cohort were classified as the group with intestinal metaplasia and (or) dysplasia and the group without intestinal metaplasia and (or) dysplasia.

### Inclusion and Exclusion Criteria

The inclusion criteria are as follows: (1) Patients with CAG who have undergone gastroscopy with pathological results showing the presence of intestinal metaplasia and (or) dysplasia; (2) Patients with CAG who have undergone gastroscopy with pathological results showing no intestinal metaplasia and (or) dysplasia; (3) CAG patients with complete clinical information.

The exclusion criteria are as follows: (1) Patients with CAG who have underwent medication in the past; (2) Patients with CAG with serious heart disease, pulmonary disease, brain disease, etc.; (3) Disabled people (blind, deaf, mute, mentally retarded, psychologically impaired, etc.); (4) Patients with CAG without complete clinical information; (5) Patients with malignant tumors; (6) Patients who are not willing to participate in this study.

### Collection of Clinical Information

Under the same evaluation process across two centers, all patients were underwent blood tests such as blood routine, blood biochemistry analysis, hemostatic function analysis, and tumor biomarker. They also underwent gastroscopy and pathological tests, which provided the information of pathology types, location, and number of the lesions, in order to determine the diagnosis and severity of the disease.

### External Validation

For external validation of the model, we recruited a total of 193 patients at the First Affiliated Hospital of Anhui Medical University, a university teaching hospital in Hefei, Anhui. We also retrospectively analyzed the clinical information of the patients in the validation group to evaluate the accuracy of the model we constructed.

### Statistical Analysis

All statistical analyses were determined using the SPSS software (version 26.0). A *t*-test was used to determine the significance of quantitative information between two groups and a chi-square test was used to determine the significance of qualitative information between two groups. Continuous variables are expressed as mean x ± SD for normally distributed variables or median (interquartile range) for non-normally distributed variables, and appropriate statistical tests (the independent samples *t*-test or the Mann–Whitney U test) were used. Categorical variables are expressed as number (n) or proportion (%) and compared using the χ^2^ test or Fisher’s exact test. Single factor logistic regression was used to determine the independent risk factors related to the prognosis of CAG patients with intestinal metaplasia or dysplasia in both training and validation cohort. Multiple factors logistic regression was conducted using variables with clinical meaning or statistical significance in the single factor analyze. A linear model was created based on the results of multiple factors logistic regression analyses. The area under the curve (AUC) of the ROC curves was used to assess the predictive accuracy of the model. The clinical usefulness and accuracy of the model was also examined by determining the net benefit using DCA curves and calibration curves.

## Results

### Results of the Single Factor Logistic Regression Analysis of the Development of Intestinal Metaplasia or Dysplasia in Chronic Atrophic Gastritis Patients

The results of the analysis based on the variables, such as general information, clinical symptoms, serologic data, and pathological data in training cohort were listed in the Table 1. A comparison of the findings in two groups showed statistically significant differences in four characteristics: gastrin-17, pepsinogen I, number of lesions, and HP infection (*p* < 0.05).

**TABLE 1:** Single factor and multiple factors logistic regression analyses of the risk factors for the onset of intestinal metaplasia or dysplasia in chronic atrophic gastritis (CAG) patients.

Characteristics	Single factor analysis	Multiple factors analysis
	*p-*value	*p*-value
Sex	0.063	
Age (yr)	0.794	
BMI	0.842	
Smoking	0.185	
Alcoholism	0.119	
Dietary pattern	0.465	
Hypertension	0.582	
Diabetes	0.458	
Helicobacter pylori (HP) infection	<0.001*	<0.001*
Number of lesions	<0.001*	<0.001*
Pepsinogen I	<0.001*	<0.001*
Pepsinogen II	0.104	
Gastrin 17	<0.001*	0.042*
CEA	0.700	
CA199	0.385	
Cholesterol	0.384	
Triglyceride	0.283	
HDL	0.225	
LDL	0.642	
WBC	0.271	
Neutrophil	0.170	
Lymphocyte	0.957	
Monocyte	0.516	
RBC	0.347	
HB	0.611	
PLT	0.390	
NLR	0.120	
PLR	0.670	
LMR	0.483	

**Ps:** We used ELISA to measure serum pepsinogen and gastrin levels. The normal range for pepsinogen I is 70–165 μg/L; The normal range for pepsinogen II is 3–15 μg/L; The normal range for gastrin 17 is 1–15 μg/L. *This data is statistically significant.

### Results of the Multiple Factors Logistic Regression Analysis of the Development of Intestinal Metaplasia or Dysplasia in Chronic Atrophic Gastritis Patients

The indicators that were statistically significant in the previous analysis were included for a multiple factors logistic regression analysis. The results showed that gastrin-17, pepsinogen I, number of lesions, and HP infection were independent risk factors of intestinal metaplasia or dysplasia in CAG patients (*p* < 0.05). The detailed results are shown in Tables 1, 2.

**TABLE 2:** The detail information of multiple factors logistic regression analysis of the risk factors for the onset of intestinal metaplasia or dysplasia in CAG patients.

Indexes	β	s_x_	Walds	*P*	95% CI
C	–0.146	0.324	0.202	0.653	–
HP infection	1.759	0.249	49.925	<0.001	[3.566, 9.463]
Pepsinogen I	–0.011	0.003	14.567	<0.001	[0.983, 0.994]
Gastrin-17	–0.043	0.021	4.125	0.042	[0.919, 0.998]
Number of lesions	2.028	0.285	50.701	<0.001	[4.348, 13.279]

### Establishment of Prediction Model and Analysis of Predictive Efficiency

Based on the above results, the mathematical equation of the prediction model is as follows:


$$Logit\left(P\right)=-0.146+1.759x_{1}-0.011x_{2}-0.043x_{3}+2.028x_{4}$$

In this equation, variable x_1_ represents HP infection (No = 0, Yes = 1), variable x_2_ represents pepsinogen I, variable x_3_ represents gastrin-17, and variable x_4_ represents number of lesions (Single = 0, Multiple = 1).

**FIGURE 1:**
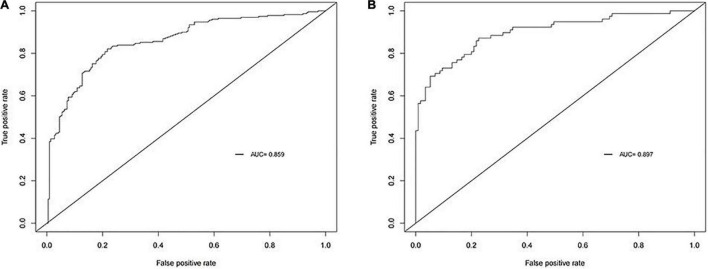
Receiver operating characteristic curve (ROC) of our model. The area under curves (AUC)are 0.859 and 0.897 in training cohort and validation cohort respectively. **(A)** Training cohort. **(B)** Validation cohort.

Next, we plotted a ROC curve based on the results we obtained. The AUC of the model were 0.859 in the training cohort (Figure 1A). Thus, the model showed great accuracy in predicting the development of intestinal metaplasia and dysplasia in patients with CAG.

**FIGURE 2:**
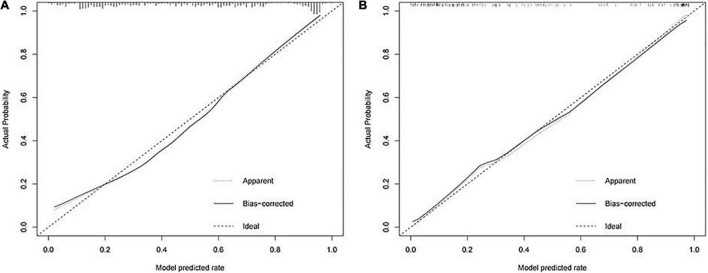
Calibration curves for predicting the onset of intestinal metaplasia and dysplasia in chronic atrophic gastritis (CAG) patients. **(A)** Training cohort. **(B)** Validation cohort.

**FIGURE 3:**
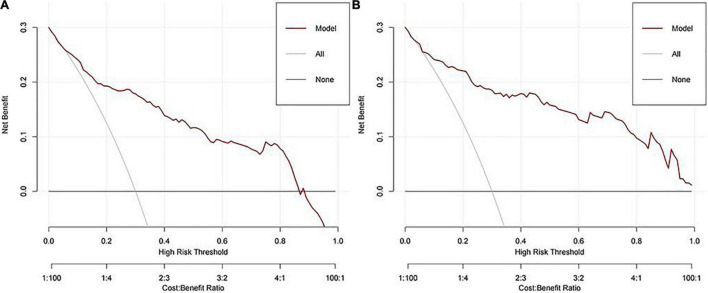
Decision curve analyses (DCA) of our model. **(A)** Training cohort. **(B)** Validation cohort.

A calibration curve and a DCA curve were also established to assess the predictive efficiency of the model based on the data in training cohort (Figures 2A, 3A). The above results indicate that the prediction model constructed in this study has a good fit and high predictive efficiency.

### Evaluation of the Prediction Model

In order to validate the predictive probabilities of the model, 193 patients recruited from the First Affiliated Hospital of Anhui Medical University were identified as validation cohort. After analyzing, The AUC of the model was 0.897 in the validation cohort (Figure 1B). A calibration curve also showed a great accuracy of the prediction model (Figure 2B). In Figure 3B, a DCA curve graphically showed that the use of the model when the threshold probability ranged from 0.1 to 0.9 added more net benefit.

## Conclusion

In conclusion, our study is the first to constructed and validate a novel prediction model based on independent risk factors to predict the onset of intestinal metaplasia and dysplasia in patients with CAG. The model is easy to apply, highly accurate, and shows great calibration. Based on this model, timely interventions can be provided to treat patients with CAG at high risk, thus improving their quality of life.

## Discussion

Numerous studies have confirmed that intestinal metaplasia and dysplasia is an important factor in the development of gastric cancer in patients with CAG (4–6). It has been found that the larger the lesion, the higher the prevalence of gastric cancer (7). Early prevention and treatment of CAG are particularly important due to its high morbidity and high risk of carcinogenesis. The impact of various risk factors on the development of CAG is different, so it is necessary to identify the influencing factors that are closely related to the disease. This study assessed the risk of intestinal metaplasia and dysplasia in patients with CAG by constructing a prediction model in order to provide a basis for early diagnosis and timely treatment of intestinal metaplasia in patients with CAG.

There are two types of CAG, type A and type B (8, 9). Type A CAG is known as autoimmune gastritis, where the main lesion is located in the body of the stomach. Due to autoimmune dysfunction, immune cells attack the parietal cell of gastric mucosa, resulting in atrophy and reduction of gastric mucosal glands, the patients therefore have a less gastric acid level. In addition, anemia may occur as a result of reduced intrinsic factors. Type B CAG is not an immune disease and its pathogenesis is associated with duodenogastric reflux, bacterial infection, and other chemical and physical damage. The main lesions are located in the gastric sinus and the patients have a normal level of acid secretion. The pathological type of CAG patients who develop precancerous lesions such as intestinal metaplasia and dysplasia has been confirmed to be type B mostly, and the pathology takes decades or more to develop. In China, the location of the lesions in patients with CAG is mainly in the gastric sinus and rarely in the body of the stomach (10).

Our study indicated that for CAG patients with intestinal metaplasia and (or) dysplasia, HP infection, a higher number of lesions, and lower serum G-17 and PG I levels were significant predictors. The factors combined with pathological, microbiological, and serological data can quantify the prognosis of CAG patients in a concise and intuitive way.

Pepsinogen is a precursor of pepsin, a gastrointestinal hormone secreted by the main cells of the stomach. It can be divided into two subtypes according to its biochemical properties and immunological activity: PG I and PG II. PG I can effectively reflect the acid secretion function of gastric mucosa, and the morphology of gastric body and the level of gastric acid secretion is positively correlated with the level of PG I (11). The reduction of gastric glands can cause a decrease in serum PG I level, therefore its level is a good predictor of CAG (12–14). Gastrin-17 is a gastrointestinal hormone secreted by gastric G cells. It can effectively reflect the secretion function of gastric sinus, indicating the degree of atrophy of gastric sinus mucosa. PG I and PGR levels were found to be significantly lower in patients with CAG and gastric cancer (GC) compared to normal (*p* < 0.01), and the levels of PG I, PGR, and G-17 are strongly correlated with the grading of CAG and the location of lesions (15). As the severity of the disease rises, the level of PG expression decreases (16, 17). Patients with lesions located in the gastric body had lower levels of PG I and PGR and higher levels of G-17, and those with lesions located in the gastric sinus had lower levels of G-17. In contrast, G-17 levels were significantly higher in GC patients (*p* < 0.01), and PG I and PGR levels were significantly lower in those with advanced GC than in those with early GC. The combined test of PG I, PG II, and PGR is of great clinical value for the diagnosis and prevention of CAG and GC. However, some studies have also found no significant difference in PG I level between the CAG and GC groups, and these scholars have suggested that PG II is a potential predictor of CAG and even GC. The result of a study on the diagnostic value of serum G-17 and PG for early diagnosis of GC in East China revealed that there were significant differences in serum G-17, PG II, and PGR levels between the non-atrophic gastritis group (NAG), chronic atrophic gastritis group (CAG), intraepithelial neoplasia group (IN), and gastric cancer group (GC; *p* ≤ 0.001) (18). The levels of serum G-17 and PG II in the IN and GC groups were higher than those in the NAG and CAG groups, while the levels of serum PGR were lower than those in the NAG and CAG groups (*p* ≤ 0.001), indicating that the serum PG I, PG II, and G-17 levels can effectively reflect the condition of the patients’ gastric mucosa and help to improve the diagnosis rate of precancerous lesions and early GC.

*Helicobacter pylori* (*H. pylori*, HP) is the most common bacteria in the gastrointestinal system and an important factor in the pathogenesis of various digestive diseases. The main pathogenic mechanisms of HP are apoptosis of the gastric mucosa due to the overexpression of self-antibodies and damage to the gastric mucosa caused by self-produced toxins (19, 20). These factors lead to atrophy of the gastric mucosa, intestinal metaplasia, and dysplasia, which ultimately cause the development of GC. HP is a risk factor for the development of CAG, several studies have indicated that people with HP infection are at higher risk of CAG (21). A systematic review of the association between HP and GC showed that the incidence of GC was 2.5 times higher in HP-infected participants than in uninfected ones (22). A 5-year study in Germany on the morbidity and risk factors of CAG found that advanced age and HP infection were key risk factors for the development of CAG (23). In addition, HP infection can cause chronic inflammation of the gastric mucosa and affect the secretion of PG and gastrin, accelerating the progression of CAG (24–26). Fortunately, timely detection and eradication of HP can effectively slow down the progression of CAG and prevent the occurrence of GC. Theoretically, the elimination of atrophy of glands and intestinal metaplasia can effectively prevent the development of intestinal type GC (27, 28). It has been found that since HP infection is the most important risk factor of atrophy of glands and intestinal metaplasia, the progression of the disease can be slowed down by eradicating HP (29). A 10-year prospective study on the association of HP eradication with the development of atrophy and intestinal metaplasia in stomach showed significant improvements in gastric sinus and body atrophy and intestinal metaplasia in patients without HP after treatment during regular track visits (30). Several studies have found that HP eradication can reverse atrophy and intestinal metaplasia, but some scholars suggest that intestinal metaplasia may be the “irreversible point” in the development of GC, and that HP eradication can only reverse atrophy, not intestinal metaplasia (29, 31–33). Although it remains controversial whether HP eradication can reverse intestinalization, many studies have confirmed that timely and effective HP eradication can reduce the morbidity of GC (34, 35).

Dyslipidemia can lead to a variety of diseases, including GC (36). Triglycerides (TG) are a risk factor for CAG. A recent study on the relationship between the triglyceride-glucose (TyG) index and the onset of precancerous lesions of GC and GC found that of 127,564 patients recruited, 43,525 (34.1%) and 186 (0.1%) were diagnosed with precancerous lesions and GC, respectively (37). What is more, patients in both the GC and precancerous lesion groups had a higher TyG index than the control group (*p* < 0.01), and there was a positive association between an increasing TyG index and the development of GCr and its precancerous lesions. Sun et al. found that high TG and TG/HDL-C ratios were significantly associated with poor prognosis in GC patients, which could be important predictors of overall survival in patients (38).

Moreover, this study is a retrospective study with inherent defects because of the potential biases, and more prospective validations are required to confirm the predictive value of our findings.

## Data Availability Statement

The original contributions presented in the study are included in the article/supplementary material, further inquiries can be directed to the corresponding author.

## Ethics Statement

Ethical review and approval was not required for the study on human participants in accordance with the local legislation and institutional requirements. Written informed consent from the patients was not required to participate in this study in accordance with the national legislation and the institutional requirements.

## Author Contributions

XL and BP: conceptualization. BP and ZW: methodology and validation. BP, ZW, QY, JW, QC, and LD: formal collection and analysis. BP: writing – original draft. XL, BP, and ZW: writing – review and editing. ZW: visualization. XL: supervision. All authors contributed to the article and approved the submitted version.

## Conflict of Interest

The authors declare that the research was conducted in the absence of any commercial or financial relationships that could be construed as a potential conflict of interest.

## Publisher’s Note

All claims expressed in this article are solely those of the authors and do not necessarily represent those of their affiliated organizations, or those of the publisher, the editors and the reviewers. Any product that may be evaluated in this article, or claim that may be made by its manufacturer, is not guaranteed or endorsed by the publisher.
